# The Oral Complications of COVID-19

**DOI:** 10.3389/fmolb.2021.803785

**Published:** 2022-01-03

**Authors:** Xinxuan Zhou, Jiajia Dong, Qiang Guo, Mingyun Li, Yan Li, Lei Cheng, Biao Ren

**Affiliations:** ^1^ State Key Laboratory of Oral Diseases, National Clinical Research Center for Oral Diseases, West China Hospital of Stomatology, Sichuan University, Chengdu, China; ^2^ Department of Pulmonary and Critical Care Medicine, West China Hospital of Sichuan University, Chengdu, China; ^3^ Department of Operative Dentistry and Endodontics, West China Hospital of Stomatology, Sichuan University, Chengdu, China

**Keywords:** COVID-19, recovery, stomatology, complications, susceptibility

## Abstract

**Background:** COVID-19 is a novel coronavirus infectious disease associated with the severe acute respiratory syndrome. More and more patients are being cured due to the development of clinical guidelines for COVID-19 pneumonia diagnosis, treatment, and vaccines. However, the long-term impact of COVID-19 on patients after recovery is unclear. Currently available reports have shown that patients recovered from COVID-19 continue to experience health problems in respiratory and other organ systems. Oral problem is one of the important complications which has serious impacts on the rehabilitation and future quality of life, such as ageusia and macroglossia, but the oral complication is often being neglected.

**Aim of Review:** From the perspective of stomatology, we summarized and elaborated in detail the types, pathogenesis of oral complications from COVID-19 patients after rehabilitation, and the reported prevention or treatment recommendations which may improve the COVID-19 patients associated oral diseases.

**Key Scientific Concepts of Review:** 1) To understand the common oral complications and the mechanisms of the development of oral complications after the COVID-19 recovery; 2) To summary the practical strategies to prevent the oral complications and construct the rehabilitation plans for patients with oral complications.

## Introduction

Corona Virus Disease 2019 (COVID-19) caused by Severe Acute Respiratory Syndrome Coronavirus 2 (SARS-CoV-2) is an acute respiratory infectious disease with a high infectivity and fatality rate. According to World Health Organization (WHO) data, by October 19, 2021, the cumulative number of confirmed cases reported globally is now over 240 million and the cumulative number of deaths is over 4.8 million.

The main clinical outcomes of COVID-19 patients are acute respiratory infection, with clinical manifestations including fever, fatigue, cough, myalgia, fatigue and other symptoms, and atypical symptoms include expectoration, headache, hemoptysis and diarrhea (Zhu et al., 2020). In clinical treatment, all patients have pneumonia, and about half had dyspnea and lymphocytopenia.

The SARS-CoV-2 can not only cause lung disease, but also a variety of systemic complications. Many patients died due to respiratory and cardiac complications (Chen et al., 2020b). Common complications in patients who died included acute respiratory distress syndrome (ARDS) (113; 100%), type I respiratory failure (18/35; 51%), sepsis (113; 100%), acute cardiac injury (72/94; 77%), heart failure (41/83; 49%), shock (46; 41%), alkali poisoning (14/35; 40%), hyperkalemia (42; 37%), acute kidney injury (28; 25%) and hypoxic encephalopathy (23; 20%). These complications caused physical injuries that were difficult to recover and seriously affected the lives of patients. In addition, the increased stress caused by COVID-19 and its complications, loss of friends or family members, damage to financial status, work stress and confinement also have a severe impact on patients’ mental state.

Angiotensin converting enzyme 2 (ACE2), targeted by SARS-CoV-2, mainly exists on the surface of human epithelial cells, especially type II alveolar epithelial cells, affecting anti-inflammatory, anti-proliferation, anti-fibrosis, anti-apoptosis of alveolar epithelial cells and vasodilator (Zou et al., 2020). ACE2 is also abundant in human oral mucosa and tongue epithelium, and SARS-CoV-2 can also bind to ACE2 receptors from oral tissues to cause the oral complications, including macroglossia, taste disorders, oral mucosa disease and so on (Aziz et al., 2020). As the entrance of digestive tract, oral cavity has a close relationship with human nutrition intake and connects the respiratory tract to assist the respiratory system. In the process of treatment of COVID-19, oral complications should be paid more attention. Meanwhile, some oral lesions may also be a warning signal for COVID-19, such as the peripheral thrombosis. Paying attention to oral lesions can start anticoagulant therapy in time to avoid more serious complications caused by peripheral thromboembolism (Favia et al., 2021).

The purpose of this paper is to summarize and discuss various oral complications of COVID-19 patients, emphasize the importance of prevention and treatment of oral complications in the rehabilitation process of COVID-19 patients, summarize the etiological mechanism to highlight the follow-up study of COVID-19 in dental practice. We also summarize the clinical diagnosis and treatment plan in depth to provide some information in the diagnosis and treatment of COVID-19.

## Taste Disorders

### Epidemiology

Taste disorders are common in COVID-19. Among COVID-19 positive patients, taste disorders are more prevalent in Europe, North America, and the Middle East, and less prevalent in Asia (Wong et al., 2020). It has been reported that the prevalence of smell and taste disorders in COVID-19 positive patients globally is 34–86% in Europe, 19–71% in North America, 36–98% in the Middle East and only 11–15% in Asia. From the perspective of gender distribution, females are more likely to suffer from taste disorders, and from the perspective of age distribution, young people are more likely to suffer from taste disorders (Lee et al., 2020).

The taste disorder can be simply divided into three types: hypogeusia, dysgeusia, and ageusia. It has been reported (Amorim Dos Santos et al., 2021) that the overall prevalence of taste disorders is 45%, of which 38% are hypogeusia, 35% are dysgeusia, and 24% are ageusia. The final statistical results are different due to different diagnostic methods in different regions and research methods including telephone, online, questionnaire and medical records review, but in general, taste disorders are a common oral complication of COVID-19 infection with a high incidence.

Due to their high incidence and some degree of presentation in the early stages of the disease, taste disorders are now accepted as a diagnostic criterion for COVID-19 in most regions. People with impaired taste are 6–10 times more likely to develop COVID-19 than people with normal taste (Wong et al., 2020). Taste disorders will alert doctors to the possibility of COVID-19 infection and need to seriously consider self-isolating and testing these people.

### Clinical Manifestations

Taste disorder is an inability to correctly perceive chemical stimuli such as taste and spiciness. Taste disorders can be roughly divided into the following three types: complete loss of taste (ageusia), reduced taste intensity (hypogeusia), distorted taste (dysgeusia) (Fjaeldstad, 2020).

The diagnosis of taste disorders can be determined by a questionnaire, which is relatively simple but subjective. Alternatively, chemical gustometry can be used.

Both COVID-19 patients and those with the acute cold can have taste disorder, but those with the disease have a harder time distinguishing bitter and sweet, while the ability to distinguish sour and salty is similar to that of those with the acute cold (Huart et al., 2020). Taste strips are also used to help us distinguish COVID-19 patients from those suffering from the acute cold.

### Hazards

As one of the five human senses, the loss of taste may seriously affect the quality of life. One effect is that patients who lose their sense of taste are more likely to develop dangerous events such as food poisoning. For example, toxic or spoiled foods often have strange tastes such as bitterness and acid, and sufferers of taste disorders are less likely to taste spoiled food. The inability to taste can lead to anorexia, which can lead to malnutrition, weakened immunity and worsening disease. People who rely on their sense of taste for work, such as chefs, bartenders and pastry chefs, may lose their jobs due to taste disorders and find it difficult to do their jobs after COVID-19 recovery, thus cutting off their financial resources. Studies have shown that taste is associated with depression, but the biological mechanisms are unclear (Yom-Tov et al., 2021). More clinical studies are needed to determine the exact association, pathogenesis, and prognosis.

### Pathogenicity Mechanism

The pathogenesis of taste disorders is still unclear, and there are several theories.

Some studies have shown that (Deems et al., 1991; Prescott, 2012) taste disorders may be related to the damage of neuronal cells by viruses, or may be caused by central nervous system ischemia. The peripheral nervous system is affected by the novel coronavirus, as the taste buds are innervated by the cranial nerves, the related functions may be impaired, resulting in taste disorders.

The production of taste mainly comes from taste receptors on the tongue, and sialic acid can protect glycoproteins responsible for the molecular transport of taste stimulation in the taste pore (Witt and Miller, 1992; Pushpass et al., 2019). Loss of taste may be due to SARS-CoV-2 binding to sialic acid receptors, which may be responsible for taste disorders. SARS-CoV-2 interactions with taste components and ACE2 receptors also have a direct impact on COVID-19-related taste disorders (Amorim Dos Santos et al., 2021).

Because taste and smell disorders often occur simultaneously, some research suggests that taste disorders may be related to the loss of smell, because the brain sometimes combines smell and taste together (Prescott, 2012). But this claim is controversial because some patients with the taste disorder COVID-19 do not develop inflammation of the nasal mucosa. Many COVID-19 patients who do not have a sense of smell disorder also develop a taste disorder (Ibekwe et al., 2020). In some patients with both taste disorder and smell disorder, the taste disorder may appear before smell disorder (Ibekwe et al., 2020).

Recent studies have also shown that taste cells can be induced to produce a large number of inflammatory cytokines, such as interleukin 6, under inflammatory stimulation (Cazzolla et al., 2020). Elevated levels of inflammatory cytokines can cause cells in taste buds to die and inhibit cell renewal, which can also lead to taste disorders (Cazzolla et al., 2020).

### Treatment

Most taste loss patients will recover within 3 weeks, and most recovery time is 7 days (Lee et al., 2020). It shows that most mild COVID-19 patients can recover their subjective sense of taste without direct medical intervention (Levy, 2020). However, nearly a year after COVID-19 patients were cured, some patients still did not recover these feelings. Alternatively, some patients regained their sense of taste, but their sense of taste was reversed and disordered, accounting for 29% of the patients according to the data reported in the study (Nouchi et al., 2021). In Nguyen’s study (Nguyen et al., 2021), 38.5% of patients had only partial taste recovery after 6 months, while 11.5% had no recovery at all after 6 months of treatment. Women are also less likely to be cured than men, accounting for 73.3% of patients who were not cured after 6 months.

SARS-CoV-2 is similar to other coronaviruses, exhibiting neural invasion (Hu et al., 2020). Neurostimulants acting on the nervous system, such as steroids, B vitamins and ATP, have been shown to be promising in treating taste disorders in COVID-19 patients (Okada et al., 2021). In addition, zinc may play a potential role in the treatment of taste disorders (2021). Zinc protects natural tissue barriers such as the epithelium, prevents entry of pathogens, balances the immune system and the redox system. As zinc is related to the action of carbonic anhydrase, lack of zinc will significantly reduce taste sensitivity and impair salivary secretion (Goto et al., 2000; Goto et al., 2001; Tanaka, 2002). Therefore, zinc supplementation can improve taste disorders (Heckmann et al., 2005). However, the use of zinc varies from person to person, and some patients with taste disorders have no effect after zinc supplementation (Sturniolo et al., 1992), even using very high zinc concentration (Lyckholm et al., 2012).

## Oral Mucosal Lesions

### Epidemiology

Oral mucosal lesions are also a common complication of COVID-19 patients. Compared to taste disorders, oral mucosal lesions are rarely reported.

Patients with oral mucosal lesions present with a variety of oral pathologies. The most common continuous lesions accounted for 73.85%, including ulcer 55.38%, aphtous lesions 12.31%, erosion 6.15%, followed by macula 6.15%, petechiae 4.61%, plaque 4.61%, bullae 3.08%, the least is gingival abnormalities. For example, peeling and necrotizing gingivitis accounted for 1.54%, and blister and pustules accounted for 1.54% (Egido-Moreno et al., 2021).

Among the patients with oral mucosal lesions, the most common site is tongue (52.56%), followed by palate and lip (16.67%), gingival (7.69%), buccal mucosa (3.85%), and connective site (2.56%) (Egido-Moreno et al., 2021).

### Clinical Manifestations

Oral mucosal lesions caused by SARS-CoV-2 have a variety of clinical manifestations, and the mucous membranes of the tongue, palate, lip, gingival and buccal of patients are affected, with varying amounts, colour and appearance. Specific symptoms include irregular ulcers, small blisters, petechiae, erythematous plaques, and desquamative gingivitis. In addition, these oral mucosal lesions can be co-infected with other viruses, and few patients are caused by a single virus (Chen et al., 2020d; Galvan Casas et al., 2020). Patients with oral mucosal lesions are usually accompanied by other skin lesions. Currently, the characteristics of oral mucosal lesions induced by COVID-19 are similar to those of non-COVID-19 patients, so the diagnosis should be combined with epidemiological history.

### Hazards

Oral mucosal lesions can cause pain and easily induce concurrent infection of other bacteria, fungi and viruses, such as *Candidiasis* and HSV-1 (Riad et al., 2020a; Tang et al., 2020). Oral mucosal lesions seem to develop into secondary manifestations and co-infection-related debilitating systemic conditions in patients (Amorim Dos Santos et al., 2021).

### Pathogenicity Mechanism

At present, there is controversy about whether oral mucosal lesions are directly caused by SARS-CoV-2 or are secondary manifestations.

The cells with ACE2 receptors are distributed on the tongue mucosa and salivary glands, these cells may become the host cells of the virus, thus causing inflammatory reactions in oral organs and tissues (Riad et al., 2020b; Amorim Dos Santos et al., 2021). Recently, Xu et al. (2020a) demonstrated the sensitivity of SARS-CoV-2 to tongue mucosa and salivary glands, and oral mucosa may be one of the targets of virus infection in humans. Therefore, oral mucosal lesions may be directly related to COVID-19 infection (Brandão et al., 2021).

However, some researchers believe that the oral symptoms are due to opportunistic or secondary infections caused by decreased immunity in the course of COVID-19 treatment after coronavirus infection. From a large number of reported medical records, many patients are co-infection and secondary manifestations. Patients’ immune status is altered during treatment, and many COVID-19 cases are fatal due to bacterial and fungal co-infection (Chen et al., 2020a; Zhou et al., 2020). In some patients, the suppressed immune system triggers reactivation of herpes simplex virus or varicella-zoster virus, leading to viral co-infection and illness. Meanwhile, oral mucosal lesions may also be induced by the immune system deterioration or disease treatment. The host produces cytokine storms during treatment for COVID-19, which can induce drug eruptions and lead to drug allergies and hives (Izquierdo-Domínguez et al., 2021).

The causes of oral mucosal lesions are complex, and many other non-viral factors can also lead to oral mucosal lesions. For example, during the COVID-19 pandemic lockdown, restrictions on social life can lead to work stress, anxiety about survival, inability to visit dental clinics, and possibly poor oral hygiene, all of which can lead to oral mucosal lesions (Guo et al., 2021). In order to reduce oral viral load after going out, some patients frequently use oral disinfectants such as hydrogen peroxide mouthwash, which may also cause oral ulcers (Hasturk et al., 2004; Petrescu et al., 2020). After the occurrence of oral mucosal lesions, the causes should be considered from various aspects.

### Treatment

For all COVID-19 patients with oral mucosal lesions, the affected site is usually cured within 3–21 days through local treatment and oral hygiene (Amorim Dos Santos et al., 2021). For patients suspected of co-infection, when conditions are available, *Saccharomyces cerevisiae* tongue scraping culture can be carried out to determine the patient’s fungal infection, and biopsy can also be used. Antifungal agents such as intravenous fluconazole and oral nystatin may be used in the treatment of these patients (Amorim Dos Santos et al., 2020). Some alcohol-free antibacterial mouthwashes can also be used, such as 0.12% sodium dichloroglyconate chlorhexidine or 1% hydrogen peroxide (Amorim Dos Santos et al., 2020).

At the same time, since oral mucosal lesions of COVID-19 patients are often co-infected, dentists should pay attention to the infection of patients from multiple aspects in the process of treatment and treat patients for different causes. Oral mucosal lesions can be accompanied by other skin lesions. In the treatment process, patients should be also observed for skin lesions during a comprehensive physical examination. In addition, dentists should explain to the treatment team the importance of maintaining oral hygiene to avoid oral mucosal lesions in COVID-19 patients.

## Macroglossia Disease

### Epidemiology

The incidence of macroglossia is relatively small. In a review of 210 patients with severe COVID-19 who had been admitted to intensive care unit in 14 clinical studies, only one (0.5%) developed macroglossia (Hocková et al., 2021).

### Clinical Manifestations

Macroglossia is a clinical diagnosis and its etiology is very complicated. A few cases of macroglossia had been reported before the COVID-19 outbreak. The clinical definition of macroglossia is that the tongue usually extends beyond the tooth or alveolar crest in its natural state. According to the different pathological types, macroglossia can be divided into true macroglossia and relative macroglossia. The main difference between the two is that true macroglossia has definite histopathological abnormalities, while relative macroglossia may have corresponding clinical symptoms, but its histological structure is normal. Some patients with macroglossia may develop different complications, such as keratinised tongue plaques or infection (Sharma et al., 2021). The most common cause of macroglossia in non-COVID-19 patients is congenital lingual vein malformation or lymphatic vascular malformation, which is more common in young children, while macroglossia caused by COVID-19 is more common in adults, most of whom have been on ventilators (Andrews et al., 2020; Sharma et al., 2021).

### Hazards

As the tongue grows, thickens and stiffens, the tongue cannot be retracted back into the mouth for a long time, which will make the tongue lose a lot of water and cause pain. A large tongue can cause tongue dysfunction and all kinds of complications. Patients will have speech disorders such as unclear speech, digestive disorders such as dysphagia, airway obstruction and other respiratory disorders. Severe cases will be completely unable to eat or speak (Brockerville et al., 2017).

### Pathogenicity Mechanism

At present, the occurrence of macroglossia in COVID-19 patients is still a rare phenomenon. There is no literature or definitive study showing the correlation between SARS-CoV-2 and macroglossia. Before COVID-19, macroglossia was caused by congenital diseases such as vascular malformations and muscle hypertrophy, or acquired diseases such as amyloidosis, hypothyroidism, endocrine disorders, metabolic disorders, tumor infiltration, viral infections, inflammatory diseases and trauma (Weiss and White, 1990).

One of the underlying causes of macroglossia is lymphatic congenital malformations (Yesil et al., 2015; Chen et al., 2020c). When the tongue is infected by bacteria or viruses, its immune system attacks the bacteria or viruses through the lymphatic system (Cheng et al., 2013). This swelling occurs in the lymphatic system to activate the inflammatory response. During the recovery of the normal inflammatory response, lymphatic will naturally detumescence back to the normal state. But the lymphatic swelling reflux obstacles can occur in some people with lymphatic congenital malformation (Horasanli et al., 2010). The lymphatic long-term blockage will result in the phenomenon of “giant tongue”. In addition, acquired angioedema can be caused by co-infection of viruses or bacteria, drug treatment, and idiopathic non-histamines (Vogel et al., 1986; Kutti Sridharan and Rokkam, 2021), all of which may be the cause of macroglossia in COVID-19.

A large number of COVID-19 patients are treated with mechanical ventilation (Azmy et al., 2020; Akhavan et al., 2021). It is also believed that the patients who recovered from COVID-19 are suffering from “macroglossia” because of mechanical ventilation in the prone position (Hocková et al., 2021). To help COVID-19 patients breathe normally, doctors may choose to strengthen their lungs by placing them prone to intubated breathing in a hospital bed. This treatment may exacerbate the blockage of lymph flow, which may lead to enlargement of the tongue (Hocková et al., 2021). This may also be related to the patient’s autonomic nervous function instability (Berger, 2020). Given that autonomic dysfunction is common in severe COVID-19 patients, the risk of macroglossia is likely to be further amplified when patients receive mechanical ventilation in the prone position (Dani et al., 2021).

Others believe that the etiology of macroglossia may also be genetic, because according to the current reports, the patients with macroglossia are mainly black race (Andrews et al., 2020; Azmy et al., 2020; Nuño González et al., 2021). According to epidemiological studies, the incidence of symptomatic angioedema in the black population is much higher due to a genetic predisposition to angioedema (Montinaro and Cicardi, 2020). However, since macroglossia is so rarely reported, more evidence is needed to construct the relationship between macroglossia and genetic predisposition.

### Treatment

To avoid severe water loss from the tongue, the tongue can be loosely wrapped with a wet saline gauze and then wrapped with compressed Coban from the distal end to the proximal end (Andrews et al., 2020). To relieve the symptoms of dry mouth, moisturizing mouthwash can also be used to moisturize (Sharma et al., 2021).

For patients with mild symptoms, local compression therapy can be used to subside the edema and allow smooth recovery (Andrews et al., 2020). Corticosteroids and bite blocks can also be used for conservative treatment (Andrews et al., 2020). For patients with severe macroglossia, surgery is the most effective way to remove the tongue hanging out of the mouth and unable to retract (Dolan et al., 1989). Because the tongue is so important for both taste and speech, the operation is delicate and difficult. When part of the tongue is removed, other parts of the tongue will replace the removed taste buds (Zhang et al., 2019). However, after partial tongue removal, the patient’s language ability may be affected (Andrews et al., 2020).

For most people with macroglossia, an oversized tongue can completely block the airways, leading to suffocation and sudden death (Alonso-Rodriguez et al., 2018). Patients with macroglossia need closely monitored and lifesaving measures such as tracheotomy (Junghaenel et al., 2012). Because mechanical ventilation in the prone position may be associated with macroglossia, some physicians also recommend other measures to improve oxygenation in patients with macroglossia to avoid further progression of the disease (DePasse et al., 2015).

## Other Oral Complications

In addition to the above oral complications, there are still some other oral complications, which are less reported, or indirectly related to oral.

### Loose Teeth

Some patients may experience symptoms such as tooth loss, gingival sensitivity, or gray or brittle teeth (Abdalla and Zwaid, 2021). Currently, the number of patients with this symptom is very small (Sirin et al., 2021). It may occur as the SARS-CoV-2 can irritate the gums, then cause sudden teeth loss.

### Oral Diseases in Patients With Mechanical Ventilation

Severe COVID-19 patients require long-term mechanical ventilation, and these patients will suffer from a large number of oral complications (Silva et al., 2020), such as bad breath, secretions retention in the mouth, mucosal damage, etc. Long-term intubation can easily lead to oral and laryngeal muscle injury, resulting in dysphonia and dysphagia after extubation (Castillo-Allendes et al., 2021).

## Discussion

Here we described in detail the common oral complications of COVID-19 which may be served as a kind of early warning sign of COVID-19 infection, as it can be used to screen quickly and initially for COVID-19 infection when. Taste disorders, oral mucosal lesions, and other clinical manifestations often occur in other diseases, but as oral complications of COVID-19, they will have some unique characteristics, once these characteristics are discovered, it is more necessary to arouse people’s vigilance. For example, patients with cold sometimes have symptoms similar to taste failure. In the clinical process, the two are easily confused, but due to the different etiology between COVID-19 complications and the common cold, there are some characteristics that can distinguish the two diseases in the practice of diagnosis and treatment. Patients with a taste deficit caused by COVID-19 have a harder time tasting bitter and sweet tastes than those suffering from a cold (Huart et al., 2020). Although both types of taste loss are often accompanied by smell loss. People with colds often have runny noses or stuffy noses, which are less common in patients with COVID-19, but COVID-19 patients have a worse ability to distinguish smells (Huart et al., 2020). This may be because mucosal congestion and edema are common in people with cold, but in COVID-19 patients, the virus attacks the chemosensory receptors, causing damage to receptors in pathways and higher cortical areas. In addition, senile taste bud sensory function degradation is also a common cause of taste disorders, in the process of diagnosis and treatment, the patient’s age is also considered. There are many causes of oral mucosal lesions, for example, immune diseases or fungal infections. Therefore, whether this symptom is a complication caused by the COVID-19 can be effectively judged by combining the simultaneous occurrence of cough, fever, dyspnea, and other symptoms. For macroglossia, the common clinical etiology is due to tongue tumors, such as hemangioma, lymphangioma, Beckwith-Wiedemann syndrome, etc. However, these patients are younger and can be diagnosed by pathology, CT, and other methods, while the patients with macroglossia caused by COVID-19 are mostly adults. And most of them had an important characteristic, that is, they had been seriously infected with COVID-19 and had received mechanical ventilation. These features of oral complications of COVID-19 are summarized in Table 1 for diagnostic reference.

**TABLE 1:** The guidelines for diagnosis and treatment of the oral complications of COVID-19 patients.

	Clinical manifestations	Distinguish from non-COVID-19 patients	Treatment
Taste disorders	Inability to correctly perceive chemical stimuli	Easily confused with the acute cold. Harder to distinguish bitter and sweet than acute cold patients. Often have smell loss, but runny noses or stuffy noses is uncommon	Mild COVID-19 patients can heal themselves. Others can use neurostimulants such as steroids, B vitamins and ATP. Zinc supplementation can be used, but need to pay attention to the zinc concentration
Oral mucosal lesions	Irregular ulcers, small blisters, petechiae, erythematous plaques, and desquamative gingivitis, etc. Co-infection with other pathogens. Accompanied by other skin lesions	Covid-19 patients often combining with cough, fever, dyspnea	Oral local treatment. Antimicrobial treatment. Keep oral microorganisms ecological balance
Macroglossia disease	Tongue extends beyond its natural state	Macroglossia caused by COVID-19 are mostly adults. Most were on ventilators	Avoid severe water loss. Mild patients can use local compression therapy. Severe patients need surgery. Tracheotomy can be used for patients with dyspnea

The human microbiome also plays an important role in human health. The oral cavity is a key part of the body’s microbial communication with the outside world, connecting the respiratory tract and digestive tract. In normal conditions, there exists a layer of barrier dominated by beneficial bacteria on the surface of oral mucosa tissue, which can prevent the invasion of pathogenic microorganisms and activate the human immune system to eliminate relevant pathogens and limit the pathogens remaining in the human body to the range of harmless to the human body. If the oral microecology is out of balance, the prevention of oral mucosa is easy to be breached by pathogens, and all systems of the human body will be mercilessly attacked. SARS-CoV-2 entry into the human body causes a decrease in oral microbial diversity and breaks the balance of oral microecology (Ma et al., 2021), which may make it easier for pathogens to enter the lungs from the mouth and lead to lung co-infection and may directly or indirectly affect the severity of COVID-19. The use of high doses or long-term antibiotics, especially broad-spectrum antibiotics, in the treatment of severe COVID-19 patients, often accompanied by secondary bacterial infections, undoubtedly contributes to the development of COVID-19 complications (Chen et al., 2020). The harm of the abuse of antibiotics not only includes liver and kidney, nerve, and blood system damage. More importantly, antibiotics kill or inhibit pathogenic sensitive bacteria, while other insensitive bacteria take the opportunity to turn over, a large number of growth and reproduction. The severe COVID-19 patients have significantly increased antibiotic resistance genes in their oral microbiome (Ma et al., 2021), which can lead to more severe infections. Therefore, the ecological balance of oral microorganisms should be considered when treating oral complications of COVID-19.

## Conclusion

Here we summarized various oral complications associated with confirmed and suspected COVID-19 patients. Among them, taste disorders have now been listed by the World Health Organization as a symptom of COVID-19 (World Health Organizatio, 2021). Oral mucosal lesions have also been shown to be associated with COVID-19. Oral complications can result from improper medication use, weakened immunity, vascular damage, local and systemic inflammation, and neglect of oral hygiene during COVID-19 treatment (Botros et al., 2020; Iranmanesh et al., 2021). For patients with COVID-19, the oral signs and symptoms should be paid more attention during the treatment, including taste disorders, pechymosis, Candidiasis, traumatic ulcers, HSV-1 infection, geographic tongue, thrush, etc (Arastehfar et al., 2020; Xu et al., 2020b; Nejabi et al., 2021). A multidisciplinary approach to oral health care and clinical oral examinations for COVID-19 patients will be benefit for the treatment and recovery. This paper highlights the importance of integrating stomatology into the COVID-19 treatment to improve the oral health and rehabilitation of COVID-19 patients (Al-Awfi, 2021).
